# High-performance graphdiyne-based electrochemical actuators

**DOI:** 10.1038/s41467-018-03095-1

**Published:** 2018-02-21

**Authors:** Chao Lu, Ying Yang, Jian Wang, Ruoping Fu, Xinxin Zhao, Lei Zhao, Yue Ming, Ying Hu, Hongzhen Lin, Xiaoming Tao, Yuliang Li, Wei Chen

**Affiliations:** 1 i-Lab, Suzhou Institute of Nano-Tech and Nano-Bionics, Chinese Academy of Sciences, Suzhou, 215123 P. R. China; 2 University of Chinese Academy of Sciences, Beijing, 100049 P. R. China; 3 Institute of Industry & Equipment Technology, Hefei University of Technology, Hefei, Anhui 230009 P. R. China; 4 Nanotechnology Centre for Intelligent Textiles and Apparel, Institute of Textiles and Clothing, The Hong Kong Polytechnic University, Hong Kong, 999077 P. R. China; 5 Beijing National Laboratory for Molecular Sciences (BNLMS), Institute of Chemistry, CAS Research/Education Center for Excellence in Molecular Sciences, Chinese Academy of Sciences, Beijing, 100190 P. R. China

## Abstract

Electrochemical actuators directly converting electrical energy to mechanical energy are critically important for artificial intelligence. However, their energy transduction efficiency is always lower than 1.0% because electrode materials lack active units in microstructure, and their assembly systems can hardly express the intrinsic properties. Here, we report a molecular-scale active graphdiyne-based electrochemical actuator with a high electro-mechanical transduction efficiency of up to 6.03%, exceeding that of the best-known piezoelectric ceramic, shape memory alloy and electroactive polymer reported before, and its energy density (11.5 kJ m^−3^) is comparable to that of mammalian skeletal muscle (~8 kJ m^−3^). Meanwhile, the actuator remains responsive at frequencies from 0.1 to 30 Hz with excellent cycling stability over 100,000 cycles. Furthermore, we verify the alkene–alkyne complex transition effect responsible for the high performance through in situ sum frequency generation spectroscopy. This discovery sheds light on our understanding of actuation mechanisms and will accelerate development of smart actuators.

## Abstract (web summary)

Transduction efficiency in electrochemical actuators hardly exceeds 1% because the current electrode materials do not allow to efficiently exploit microstructural changes to achieve large actuation effects. Here the authors demonstrate transduction efficiencies up to 6% in actuators by using graphdiyne as electrode material.

## Introduction

Bio-inspired artificial muscles, actuated in response to external stimulus, have attracted intensive attention in bionics, including robotics, intelligent sensors, and micro electro-mechanical systems during the past decades^1–3^. Electroactive polymer (EAP) actuators have become the priority research area of artificial muscles by virtue of their lightweight, scalability, low-power dissipation, quick response, and large deformation^4–6^. Ionic polymer metal composite (IPMC) actuators, also called electrochemical actuators, have emerged as one of the most attractive EAP due to their superior performance and cycling stability under low driving voltage^7–13^. As the electromechanical stain of IPMC actuators is generated by the reversible ion intercalation and deintercalation in electrodes, the ion storage capacity of electrodes becomes crucial to actuators^14,15^. Strangely, higher ion storage often leads to higher energy storage capacity of electrodes, but does not always lead to higher actuation performance. Although high-energy-storage electrode materials, especially nanocarbon materials, have emerged one after another, highly electro-mechanical transductive actuators have been seldom reported till now^16–18^.

As actuation performance is mainly dominated by the electrochemical and electromechanical processes of the electrode layer, figuring out the mechanism and dynamics process in the electrode is essential to developing next-generation actuators with higher performance. Hitherto, there are two kinds of electrochemical actuation mechanisms that are widely accepted. One mechanism, called the quantum-mechanical effect, was proposed by Baughman in 1999, who reported a CNT actuator with exceptional electrochemical actuation in aqueous electrolyte^4,19,20^. In 2003, Wanlin Guo further proved giant C–C bond elongation in response to charge injection into CNT structure using Hartree–Fock and density functional quantum mechanics simulations^20,21^. The other one, called electrostatic double-layer effect, was proposed for graphene actuator by Rogers^22,23,]^ in 2011, who made detailed theoretical calculations to prove its rationality through density functional theory (DFT). Owing to these landmark works, various actuators with superior performance have emerged one after another, greatly promoting the development of smart material^24–29^; however, the energy transduction efficiency of these actuators is always lower than 1.0%. This is mainly because conventional pure sp^2^ carbon-based electrodes, including graphene and carbon nanotube, always lack the active units in nanostructure, and their assembly systems can hardly express the intrinsic properties but mainly rely on ion adsorption for actuation.

Graphdiyne, which features assembled layers of sp and sp^2^ hybridized carbon atoms, has been proposed to be the most stable carbon allotrope with high π-conjunction, uniformly distributed triangular pores, and tunable electronic properties^30–33^. It is believed that graphdiyne, a newcomer of carbon nanomaterials, can compete in various potential applications with conventional sp^2^ hybridized carbon systems, namely fullerenes, CNT, and graphene^34–37^. The porous structure enables both in-plane and out-plane diffusion of ions with moderate barriers^38,39^. Compared with pure sp^2^ carbon-based materials, high complexation activity is a unique property of graphdiyne within numerous molecular-scale active alkyne units. But most of the previous researches^40–43^ mainly concentrated on the high complexation activity of graphdiyne in electronic devices, photovoltaics, energy storage and catalysis, the significant strain, brought by the molecular structural change of complexation process, was neglected.

Here, we report and utilize the large strain induced by ionic complexation effect, and, surprisingly, we found that a strain as high as 16.45% can be achieved. Moreover, we fabricate an ionic polymer graphdiyne composite actuator with a high electro-mechanical transduction efficiency of up to 6.03%, exceeding that of the best-known piezoelectric ceramic, shape memory alloy, and EAP reported before, and its energy density (11.5 kJ m^−3^) is comparable to that of mammalian skeletal muscle (~8 kJ m^−3^). Meanwhile, the actuator remains responsive at a wide frequency range from 0.1 to 30 Hz and shows excellent cycling stability in air over 100,000 cycles. Furthermore, we put forward an alkene–alkyne complex transition mechanism responsible for the high performance and then verify the mechanism through in situ sum frequency generation (SFG) spectroscopy. This present study paves a way for designing electrodes with high electrical energy storage as well as high electro-mechanical transduction efficiency, and inspire later researchers to make more efforts on disclosing the energy transduction mechanism of actuators as well as other smart materials.

## Results

### Preparation and characterization of graphdiyne

**Fig. 1:**
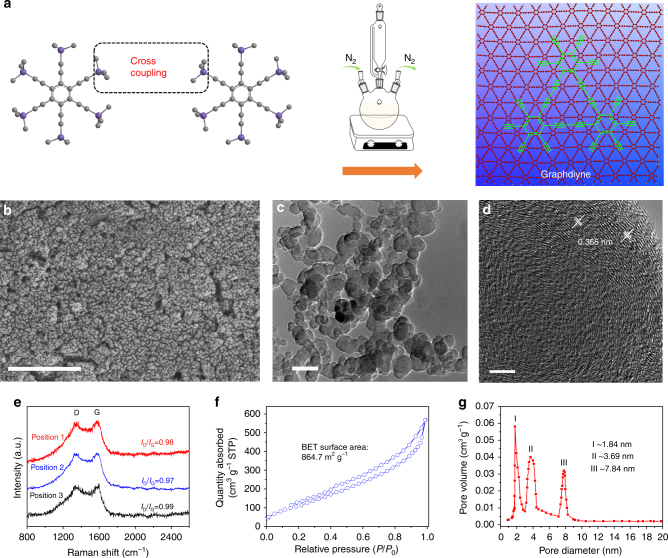
Synthesis and microstructure characterizations of graphdiyne. **a** Schematic illustration for the synthesis of graphdiyne. **b** SEM image of graphdiyne, scale bar 500 nm. **c** TEM image of graphdiyne, scale bar 100 nm. **d** HRTEM images of graphdiyne, scale bar 5 nm. **e** Raman spectra of graphdiyne. **f** N_2_ adsorption/desorption isotherms of graphdiyne. **g** Pore size distribution of graphdiyne

In a typical preparation process^44,45^, graphdiyne was synthesized on the surface of copper via a cross-coupling reaction using hexaethynylbenzene, as illustrated in Fig. 1a. The morphologies of as-synthesized graphdiyne were examined by scanning electron microscopy (SEM) and transmission electron microscopy (TEM). The SEM image in Fig. 1b shows that graphdiyne was composed of nanoparticles that aggregated together to form a hierarchical porous structure. From the TEM image in Fig. 1c, it can be clearly seen that graphdiyne was composed of nanoparticles with abundant porous structure, which is consistent with the SEM images. The morphologies of CNT and graphene are presented for comparison in Supplementary Fig. 1. By the way, CNT showed tube-like morphology while graphene showed laminar morphology. The high-resolution transmission electron microscope (HRTEM) image (Fig. 1d) of graphdiyne nanoparticle clearly shows lamellar structure with an adjacent interlayer distance of 0.365 nm, which exhibits numerous porosity and ion channels. The selected-area electron diffraction pattern in Supplementary Fig. 2 reveals typical diffraction rings and verifies its amorphous structure.

**Fig. 2:**
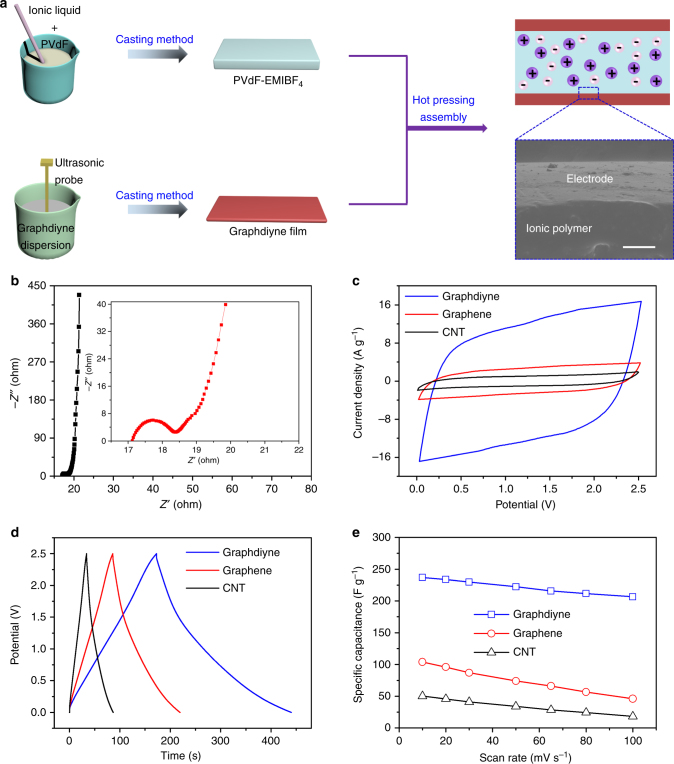
Assembling and electrochemical properties of the graphdiyne actuator. **a** Schematic for the assembly procedure of the graphdiyne actuator. Cross-sectional SEM image of the graphdiyne actuator with fine interfacial adhesion, scale bar 10 μm. **b** Nyquist plot for the device. The inset shows magnified 0–22 ohm region. **c** CV curves of the devices based on graphdiyne, graphene, and CNT at a scan rate of 100 mV s^−1^, respectively. **d** Charge−discharge curves of graphdiyne, graphene, and CNT actuators at the current density of 1 A g^−1^. **e** Specific capacitances of actuators as a function of current density

To further verify the microstructure of graphdiyne, structure characterizations, such as X-ray diffraction (XRD) and Raman spectra, were conducted. The XRD pattern in Supplementary Fig. 3 confirms that graphdiyne has amorphous structure^45–47^. The Raman spectra in Fig. 1e verify the quality and uniformity of graphdiyne powder. Peaks observed at 1349 and 1590 cm^−1^are the D (disordered and defective structure) and G (graphitic structure) bands of carbon materials, respectively^48,49^. Graphdiyne in this work was synthesized through cross-coupling reaction using micromolecules, resulting in nanoparticle morphology with more defects compared with the CVD-grown method. Thus, the defect condition of chemosynthetic graphdiyne was not as good as that of CVD-grown graphene. After optimizing the synthesis conditions, the* I*_D_/*I*_G_ band intensity ratio of graphdiyne could be controlled to 0.98 with a relatively higher graphitization degree and fewer defects^36,45,50^. In addition, peaks at 1926.2 and 2189.8 cm^−1^ can be assigned to the vibration of conjugated diine links^51,52^. As we know, specific surface area and porosity are vital to the performance of electrode materials. Figure 2f, g displays the characterization of surface area and porosity of the graphdiyne powder. The specific surface area of graphdiyne was as high as 864.7 m^2^ g^−1^ and pore size distribution (shown in the inset) was mainly around 1.84, 3.69, and 7.84 nm. The micropores, mesopores, and high surface area were expected to improve ion storage capacity and facilitate ion transfer, finally enhancing the energy storage capacity, and rate capacity of graphdiyne electrode^53^. Otherwise, the specific surface area of CNT and graphene were only 96.7 m^2^ g^−1^and 190.3 m^2^ g^−1^ with wide pore size distribution, respectively (Supplementary Figs. 4 and 5).

### Fabrication and electrochemical characterizations of the graphdiyne actuator

To evaluate the practical value of graphdiyne material, a flexible ionic actuator was constructed by laminating two pieces of electrode films with an electrolyte layer. Schematic of the assembling procedure and structure of the actuator are shown in Fig. 2a. The cross-sectional SEM image of the actuator indicated that the electrode exhibited a strong interlayer adhesion with the polyelectrolyte layer. Nyquist plot of this actuator is presented in Fig. 2b, including a small semicircle (high-frequency region), the Warburg diffusion line (middle-frequency region), and the capacitive line (low-frequency region)^54,55^. Equivalent series resistance of the device is 18.4 ohm, showing a good interface contact between the electrolyte and electrode, as well as excellent electronic conductivity of the electrode. The plot line of the low-frequency region is almost vertical to the *y* axis, indicating an ideal capacitive behavior. Moreover, we compared the Nyquist plot of graphdiyne with those of graphene and CNT in Supplementary Fig. 6 and made the electrolyte in supplementary (EIS) molding using the equivalent circuit in the inset. EIS molding data including *R*_s_, *R*_ct_, *Z*_w_, and *C*_I_ are shown in Supplementary Table 1. According to the molding results, the graphdiyne actuator showed the best electric contact, ion diffusion ability, and energy storage capacity when compared with others.

In order to evaluate the electrochemical properties of the graphdiyne actuator, cyclic voltammogram (CV) curves of CNT, graphene, and the graphdiyne actuators were compared in Fig. 2c. The CV curves all display regular rectangular shapes, demonstrating their excellent capacitive behavior. In this work, we conducted CV measurements on thin-film devices for evaluating the energy storage capability. The measurements were all conducted under PVdF/EMIBF_4_ polyelectrolyte with an electrochemical window of 2.5 V and scan rates from 10 to 100 mV s^−1^. Apparently, the graphdiyne actuator shows the highest energy storage capability with a specific capacitance of 237 F g^−1^ at the scan rate of 10 mV s^−1^, which is nearly five times that of CNT actuator. In addition, galvanostatic charge−discharge tests in Fig. 2d were measured at a current density of 1 A g^−1^ to further evaluate the energy storage performance of the devices. The typical triangular shapes confirm the good coulombic efficiency. More importantly, the specific capacitance of the graphdiyne actuator still remains at 206 F g^−1^ even at a scan rate as high as 100 mV s^−1^, revealing a better rate capability than the other two (Fig. 2e). The superior energy storage performance of graphdiyne actuator mainly results from its typical triangular pore in framework, numerous highly active alkyne sites, and hierarchical porous structure with a high specific surface area of 864.7 m^2^ g^−1^, as well as the high electrical conductivity. Specifically, the triangular pore and hierarchical structure of graphdiyne facilitate ion migration during the charge−discharge process, and the high specific surface area makes graphdiyne electrode act as the ion reservoir, which guarantees the high energy storage capability of the graphdiyne actuator, as well as rate capability^36,56^. The numerous alkyne sites played the role of an ion-attracting magnet in graphdiyne. The superior energy storage capability of the graphdiyne actuator indicates that it would be a promising candidate as a highly efficient energy transduction actuator.

### Actuation performance of low-voltage-driven electrochemical actuators

**Fig. 3:**
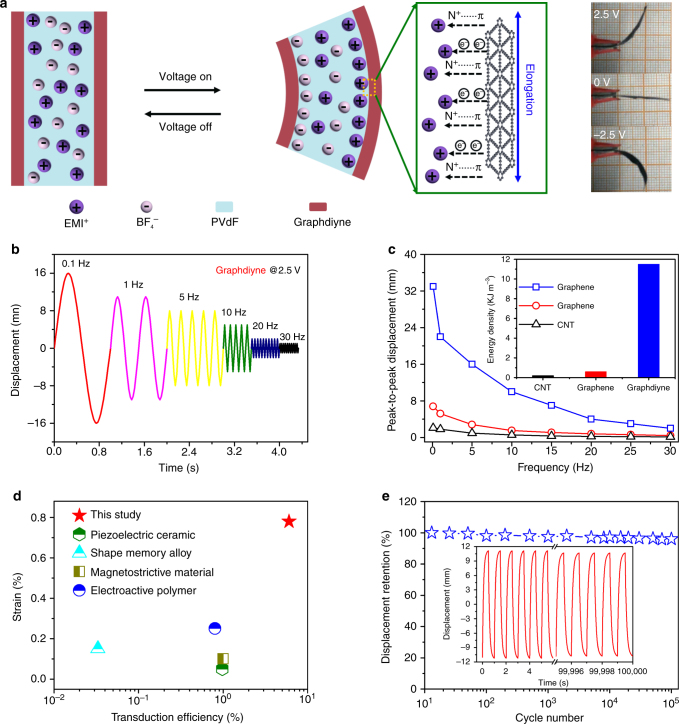
Actuation performance of the graphdiyne actuator. **a** Schematic for actuation mechanism of the graphdiyne actuator. Optical images show the bent actuator under 2.5 V at 0.1 Hz. **b** Attenuation strain of the graphdiyne actuator with increasing frequency. **c** Peak-to-peak displacement as a function of frequency of the three actuators. Inset is the comparison of their power densities. **d** Comparison of transduction efficiency of different types of actuation materials. **e** Cyclic stability of the graphdiyne actuator. Inset shows the actuation cycles

With the aim of translating this large stored electric energy into mechanical deformation, a flexible bimorph cantilever actuator (Fig. 3a) was evaluated through multi-step voltage stimulus (Supplementary Fig. 7). The graphdiyne actuator makes reversible actuation strain with the electric power on and off. Firstly, cations and anions in the polyelectrolyte immigrate to the cathode and anode, respectively, under the applied electric field. Then, cations coordinate with graphdiyne structure, leading to alkene–alkyne complex transition along with dimensional changes of the covalent bond network in cathode. Meanwhile, the anode does not exhibit such a transition effect with anions. As a result, the imbalanced elongation of opposite electrodes leads to strain of the bimorph cantilever structure.

With no doubt, the superior energy storage capacity accompanied by numerous highly active transduction units would make some breakthroughs for the graphdiyne actuator. Peak-to-peak displacements of CNT, graphene, and the graphdiyne actuators were measured at various excitation frequencies from 0.1 to 30 Hz. Displacement of the graphdiyne actuator decreases with increasing frequency at the voltage of 2.5 V (Fig. 3b). This is mainly because the time duration for ion migration into electrode is insufficient at higher frequencies^57^. Strain of the graphdiyne actuator was impressively higher than those of the other two (Fig. 3c). Moreover, the graphdiyne actuator could still maintain a relatively large strain of 0.07% at a frequency of 30 Hz, while CNT and graphene actuators lost the current response beyond 10 Hz. We evaluated actuation performances of the three actuators under 0.5 V at a frequency of 0.1 Hz and drew the input voltage and responses in Supplementary Fig. 8. With the decrease of voltage to 0.5 V, actuation displacements of all actuators fell into relatively lower values, but the graphdiyne actuator still kept the highest deformation ability of all. Because lower input voltage weakened the ion migration rate in actuators and thus led to lower actuation displacement. On the other hand, phase delay of the three actuators seems negligible according to our results above, which is also in accord with those of previous reported actuators based on CNT and graphene^58–60^. This is mainly because of the superior properties of carbon nanomaterials, such as high electric conductivity, large specific surface area, and flexible molecular structure, which are of benefit for electron conduction, ion migration, and energy transduction^3,61,62^. With regard to energy density (Inset in Fig. 3c), the graphdiyne actuator has an overwhelming advantage over CNT and graphene actuators. It is worth mentioning that the energy density of graphdiyne actuator achieved was as high as 11.5 kJ m^−3^, comparable to mammalian skeletal muscle (~8 kJ m^−3^)^63^. According to the stress−strain curve in Supplementary Fig. 9, Young’s modulus of the graphdiyne actuator is 420 MPa. Moreover, the actuation stress (3.11 MPa) generated during alkene–alkyne complex transition process of graphdiyne was significantly higher than that of human skeletal muscle (0.3 MPa)^2^. We measured the blocking force of all actuators using a load cell (JZ-101, XINHANG) under 2.5 V at a frequency of 0.1 Hz. The graphdiyne actuator showed a relatively higher blocking force of 3.37 mN than those of CNT (1.38 mN) and graphene (1.92 mN) actuators, and we will improve its level in our future work.

Ultimately, the actuation performance of an electrochemical actuator depends on the electro-mechanical transduction efficiency within the material. Thus we evaluated the transduction efficiency according to a simple but reliable method reported by Cottinet et al.^64,65^. The calculated efficiency of the graphdiyne actuator was as high as 6.03%, which exceeds that of any alternative actuator material^66–68^, such as piezoelectric ceramics, shape memory alloy, magnetostrictive material, and EAP (Fig. 3d). To gain insight into the high performance of the graphdiyne actuator, the contribution of charge injection to the actuation strain of different actuators (Supplementary Fig. 10) was evaluated by referring to previous theoretical study^21,22^. The dependence of strain on charge injection for covalent carbon materials is quasi-parabolic, consistent with the theoretical results. Obviously, graphdiyne actuator demonstrates the highest strain of the three actuators under the same charge injection. This implies that the electrochemical strain induced by alkene–alkyne effect is impressively higher than those induced by quantum-mechanical effect or electrostatic double-layer effect under the same electric stimulus. The graphdiyne actuator also exhibits excellent cycling stability with little strain degradation after 100,000 cycles (Fig. 3e). A comparative table on the actuation properties of graphdiyne with graphene, CNT, and other materials is presented in Supplementary Table 2. It is worth mentioning that the graphdiyne actuator in this work showed a higher energy transduction efficiency compared with other materials, resulting from the highly active molecular-scale actuation mechanism.

Then, we assembled the graphdiyne actuators based on PVA-H_2_SO_4_ and PVA-KOH for comparing with the ionogel-based system in Supplementary Fig. 11. According to the results, conventional gel actuators including PVA-H_2_SO_4_ or PVA-KOH systems could only work below the actuation voltage of 1.0 V, limited by the low electrochemical window of water molecules. Moreover, conventional gel actuators could not remain stable because of water loss in air. However, ionogel-based actuators did not have these two problems because of the air stability and nonvolatility of ionic liquid^11,69,70^. We controlled the thickness of graphdiyne films by changing the dosage of casting solution and evaluated its influence on the performance of the actuator in Supplementary Fig. 12. Because of the limitation of the solution-casting method, the thinnest graphdiyne film we could fabricate in this work was 12 μm because if we continued to decrease the dosage of the casting solution the resultant graphdiyne film could not be freestanding. According to the result below, actuation displacement decreased with the increasing thickness of the graphdiyne film. This is because that a thicker graphdiyne film will increase the bending resistance, which needs to be overcome during the actuation process; thus a thicker electrode film always leads to damped actuation strain. To demonstrate the versatility of the graphdiyne actuator, we developed three bio-inspired models (Supplementary Fig. 13) by virtue of their high performance. A flying robot model with two graphdiyne actuator wings was fabricated utilizing its high-frequency responsiveness. The fast and visible motion of the flying robot indicated its great potential for biomimetic insects. Also, we designed and fabricated another two useful examples, wiper and crane models, by employing the actuators.

## Discussion

**Fig. 4:**
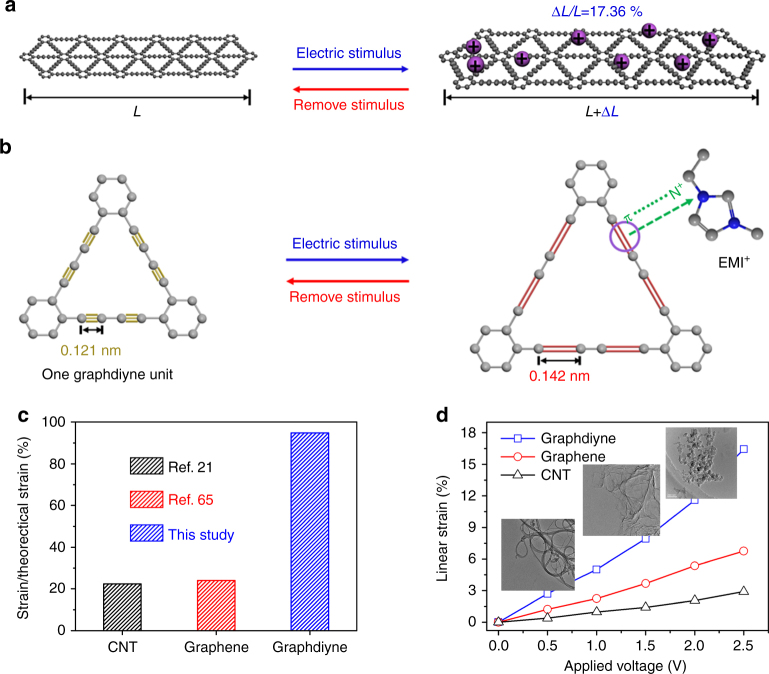
Actuation mechanism of graphdiyne materials. **a** Schematic for actuation strain of graphdiyne when encountering electric stimulus. **b** Alkene–alkyne complex transition mechanism in one graphdiyne unit. **c** Linear strain of graphdiyne, graphene, and carbon nanotube films under various applied voltages by SECM method. Inset shows TEM images of the three materials. **d** Strain retention of theoretical value of the three materials

**Fig. 5:**
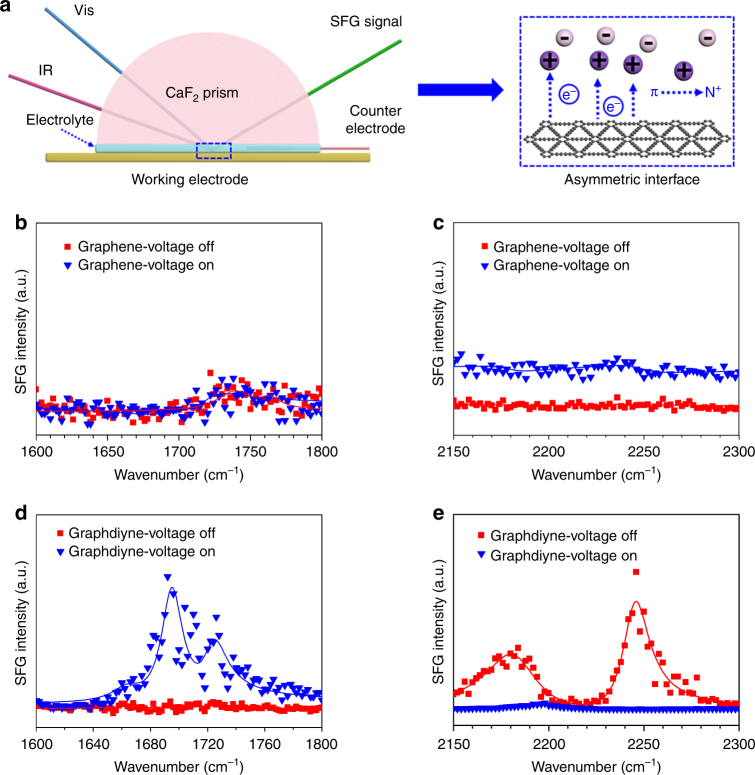
Verification of alkene–alkyne complex transition mechanism through the SFG method. **a** Schematic of the SFG simulative cell. The technique is a second-order nonlinear process with inherent interface selectivity. It involves the overlap of two pulsed laser beams at the asymmetric interface, generating a new beam with a sum frequency of the two. **b**, **c** SFG signal of the graphene electrode/EMIBF_4_ upon voltage on and off at alkene bond stretching region (**b**) and alkyne bond stretching region (**c**). **d**,** e** SFG signal change of the graphdiyne electrode/EMIBF_4_ upon voltage on and off in the alkene bond stretching region (**d**) and alkyne bond stretching region (**e**)

In light of the outstanding performances obtained above, we put forward an alkene–alkyne complex transition mechanism of graphdiyne materials. Figure 4a, b shows the schematics for this mechanism in a single layer and one structured unit, respectively. Specifically, surrounding ions (1-ethyl-3-methylimidazole cations) absorbed onto one graphdiyne unit under electric stimulus and then coordinated with alkyne bonds; because of their unoccupied orbitals, alkyne bonds would transform into alkene bonds accompanied with variation of bond length. This bond length variation of alkene–alkyne complex transition finally leads to dimensional change of covalent bond network in graphdiyne structure. Moreover, the transition process is reversible and controlled by external electric fields. The theoretical strain of graphdiyne during the complexation process was evaluated by its bond length change ratio according to previous works^13,71^. Surprisingly, we found that a strain as high as 17.36% can be achieved (bond lengths were obtained from the simulating result of ChemBioOffice software of Cambridge Soft Corporation, USA). As for energy storage process, the numerous alkyne sites with high ion complexation activity, which was verified by in situ SFG method in Fig. 5, greatly enhanced the binding interaction of graphdiyne with cations and increased the charge density of electrode during electrochemical charging and discharging process. More importantly, the elongation strain induced by alkene–alkyne transformation provided more interspace for ion migration and thus improved the energy storage capacity greatly in Fig. 2.

To verify the theoretical prediction above, we fabricated a freestanding graphdiyne film through solution-casting method and found that this film could be rolled around a glass rod with excellent flexibility (Supplementary Fig. 14). Then, we studied its electrochemical strain through scanning electrochemical microscopy (SECM). The imaging mechanism is based on the feedback current of the microelectrode probe, which varies with the distance between the probe and working electrode (Supplementary Fig. 15). Thus we performed in situ monitoring of the actuation process of graphdiyne film by utilizing this technique. We designed a scratch method to detect the linear strain of CNT, graphene, and graphdiyne electrode films (Supplementary Fig. 16). The scratch marked on the film would contract after encountering electric stimulus and the strain was evaluated by contraction ratio of the scratch. As shown in Fig. 4c, graphdiyne electrode shows the highest deformation ability (strain of 16.45% under 2.5 V) of the three throughout the voltage range. The actuation processes of CNT and graphene materials mainly rely on comparatively inefficient physical mechanisms, such as quantum-mechanical mechanism and electrostatic double-layer mechanism, but graphdiyne actuation depends on active alkyne sites with more highly efficient transition mechanism. Notably, the experimental strain of graphdiyne film is close to the theoretical value with a strain retention of 94.8% in Fig. 4d, but the experimental value of CNT and graphene film cannot achieve the theoretical level reported before^21,62^. According to references reported before^59,69,72^, intrinsic graphene actuators mainly rely on the capacitance mechanism, which means higher ion adsorption always leads to larger actuation strain because of the larger expansion of cathode. Sulfur and nitrogen co-doped graphene electrode^57^ was also designed to further enlarge this capacitive effect because of the pseudo capacitance. Essentially, previous reported electrodes, such as noble metals and carbon nanomaterials with a sp^2^ hybrid system, mainly rely on the capacitive effect for actuation without any molecular-scale electro-mechanical activity. The appearance of graphdiyne with a special sp and sp^2^ co-hybrid system provided an opportunity to improve the performance of next-generation actuators because graphdiyne not only had high ion capacity because of its higher surface area and abundant pores, but also showed molecular-scale electro-mechanical activity based on alkene–alkyne complex transition effect. As this molecular-scale electro-mechanical activity was hard to detect by conventional characterizations, we used in situ SFG spectroscopy with high interface selectivity and single-molecule layer sensitivity to verify this effect, which has never been reported before. Thus the graphdiyne actuator showed excellent actuation performance, such as high energy transduction efficiency, muscle-comparable energy density, and long cycle life.

In order to verify the alkene–alkyne complex transition mechanism, in situ SFG spectroscopy was used to probe the interfacial interactions (Fig. 5a) between graphdiyne and ions by applying a constant voltage to drive the ionic complexation process. A simulated cell was constructed for SFG tests, comprising graphdiyne film with 1-ethyl-3-methylimidazole tetrafluoroborate (EMIBF_4_) electrolyte. Photographs of SFG cell are also presented in Supplementary Fig. 17. The SFG technique is based on a second-order nonlinear optical effect, which can be utilized to in situ monitor the dynamic process of interface molecules. When two pulsed laser beams irradiate to the interface of different materials, it would then generate a new beam with a sum frequency of the two input beams. The SFG technique possesses inherent interface selectivity owing to the fact that even order susceptibilities become zero in centrosymmetric media under electric dipole approximation. The SFG intensity from an interface region is proportional to the incident laser intensities and the square of the absolute value of the effective sum frequency susceptibility.

Obviously, there is no SFG signal response in graphene electrode/EMIBF_4_ with or without the voltage (Fig. 5b, c). This is because pure sp^2^ carbon materials, including CNT and graphene, lack active units in the structure and do not have any more interaction with surrounding ions except electrostatic force. As for graphdiyne electrode/EMIBF_4_ system, the situation is quite different. After applying a voltage of −2.5 V onto the electrodes, alkene peaks of graphdiyne at around 1695 and 1725 cm^−1^ started to appear with time elapsing (Fig. 5d), while the alkyne peaks at 2180 and 2246 cm^−1^ gradually disappeared (Fig. 5e). Analysis of peak assignments is shown in Supplementary Note 1 and 2. This result implies that alkyne bonds have transformed into alkene bonds when the surrounding ions coordinate with the active units under the applied voltage. In addition, the disappeared alkyne peaks and the arisen alkene peaks both returned to their original states after removing the voltage (Supplementary Figs. 18 and 19), reflecting the reversibility of the transition. Notably, this transformed alkene bond was slightly different from conventional double bond because electrons on the alkyne bond did not completely transfer to the coordinated ions, but just skewed toward them under electric field. Thus the transition process becomes reversible with electric stimulus on and off. Finally, the complexation-assisted alkene–alkyne complex transition was verified through SFG characterizations and would assure us much expectation for applying this material in actuators. We also conducted additional experiments about in situ SFG measurement in the aqueous PVA–KOH system, but did not observe the mechanism of alkene-alkyne transformation in Supplementary Fig. 20. As we know, cations showed hydration effect, which was competitive with the complexation with graphdiyne electrode in aqueous gel electrolyte. This saturated hydration effect was much stronger than the complexation effect. But ionogel electrolyte did not show such hydration effect; thus, the alkene-alkyne transformation effect in this system was dominant and easy to detect by in situ SFG instrument. As a result, the actuation performance of the graphdiyne actuator with ionogel was much better than that with aqueous gel EIS Fig. 11. As the stainless steel parts of SFG cell were susceptible to corrosion in sulfuric acid solution, we did not conduct a control test in PVA-H_2_SO_4_ solution, but the result would be the same as that in PVA-KOH solution because of the same hydration effect.

In summary, we put forward and verify an alkene–alkyne complex transition mechanism for graphdiyne materials in this work. Based on the highly efficient actuation mechanism, the as-fabricated the graphdiyne actuator displays intriguing actuation performances, including quite high frequency response (strain of 0.07% at 30 Hz), high energy transduction efficiency (6.03%, exceeding that of the best-known actuation materials), large energy density (11.5 kJ m^−3^, comparable to mammalian skeletal muscle), and excellent cycling stability in air (negligible degradation after 100,000 cycles at 1 Hz under 2.5 V). Moreover, the fast and visible motion of the three robot models indicated its great potential for biomimetic devices. Finally, and most remarkably, the alkene–alkyne complex transition mechanism not only reinforced actuation performance, but also gave a perspective on ionic complexation for researchers. We anticipate that this discovery will present an insight into the rational design of electrode materials for other electrochemical devices, such as mechanical sensors and lithium-ion batteries.

## Methods

### Synthesis of graphdiyne

Copper foils were sonicated with hydrochloric acid (HCl, 4 mol L^−1^, 100 mL) for 3 min, washed with water and ethanol with sonication for 3 min, and then washed with acetone (2 times) before drying under nitrogen (N_2_). Many pieces of copper foil (2 × 2 cm^2^) were immersed into 50 mL pyridine and then heated to 120 °C under N_2_ for 1 h. After that, the mixture was cooled down to 80 °C for next use. Hexakis[(trimethylsilyl)ethynyl]benzene with the mass of 20 mg was dissolved in 50 mL tetrahydrofuran (THF) with a bath of ice and ammonium chloride under N_2_ atmosphere for 30 min. A solution of tetra-*n*-butylammonium fluoride (TBAF) in THF (1 mol L^−1^, 2.5 mL) was added into the mixture and then stirred for 15 min at low temperature under N_2_ atmosphere. If the solution had a purple color, its quality was regarded as high. The reaction mixture was blended with ethyl acetate, washed with saturated sodium chloride solution (NaCl, 3 times), then dried with magnesium sulfate (MgSO_4_), and lastly filtered. The filters were dried under vacuum with a temperature below 30 °C in dark environment. The product was dissolved in 50 mL pyridine and then added dropwise into the 50 mL mixture of pyridine with copper foil at 80 °C under N_2_ protection for 8 h. After dropping, the reaction system was kept for at 120 °C for 3 days. After completion of the reaction, the solvent was removed by vacuum distillation with black films on the copper foils. The foils with graphdiyne films were then washed with acetone, hot dimethylformamide (DMF, 80 °C), and ethanol, respectively. Finally, copper foils with graphdiyne films were dried at 100 °C under vacuum for 1 h. The graphdiyne powder was collected with the yield of 71.5%.

### Synthesis of PVdF/EMIBF_4_ electrolyte layer

Firstly, 1.0 g PVdF and 1.5 g EMIBF_4_ were dissolved in 20 mL DMF at 30 °C. Then, the solution was poured onto teflon mold and kept at 60 °C for 2 days. Finally, an electrolyte film (size 75 mm × 25 mm) was obtained by peeling off from the mold.

### Synthesis of electrode film and fabrication of actuator

Firstly, 70 mg electrode materials (CNT, graphene or graphdiyne powder) and 30 mg PVdF were dispersed in 20 mL DMF for 30 min through horn sonication treatment in an ice-water bath. Subsequently, the suspension was casted onto a mold and became a freestanding electrode film (size 75 mm × 25 mm) after drying at 70 °C for 9 h. Lastly, the actuator (25 mm × 2.5 mm × 80 μm) was assembled by laminating two pieces of the as-prepared electrode films with PVdF/EMIBF_4_ electrolyte separator through the hot pressing method.

### Characterization techniques

SEM and TEM images were recorded by Hitachi S-4800 and FEI Tecnai G2 F20, respectively. Raman spectra were conducted with a LabRAM HR800 from JY Horiba. X-ray photoelectron spectra were obtained using PHI 5000 VersaProbe II instrument. X-ray powder diffraction analysis was measured by Philips X’Pert PRO diffractometer with nickel-filtered Cu *Kα* radiation. Nitrogen sorption analysis was done with Micromeritics ASAP 2020 instrument using the Brunauer–Emmet–Teller method and the pore size distribution plots were evaluated based on the DFT. In situ SFG spectrometer laser system (PG 500) was built by EKSPLA Company in Lithuania, using a copropagating configuration. SFG cell was custom-made from Tianjin ida Science and Technology Co. Ltd in China.

### Electrochemical measurement and actuation test

All the electrochemical characterizations were recorded by a CHI760D electrochemical work station. The specific capacitance (*C*) was calculated at various scan rates from 10 to 100 mV s^−1^ by using the following equation^59,73^:

1 $$C \,({\mathrm{F}}\,{\mathrm{g}}^{ - 1}) = \frac{{{{S}}_{\mathrm{Area}}}}{{2{{v}} \cdot {\mathrm{\Delta }}}V \cdot m}.$$

Here, $S_{\mathrm {Area}} = \mathop {\oint }\nolimits I \mathrm {d}V$ is the loop area of the CV curve, *v* (V s^−1^) is the scan rate, Δ*V* (V) refers to the potential window, and *m* (g) presents the mass of active material in the electrode.

All SECM measurements were conducted with a CH Instruments model CHI 900 SECM. The linear strain (*ω*) of electrode films was calculated using the following equation^13^:

2 $$\omega = \frac{{W_1 - W_2}}{{W_1}} \times 100{\mathrm{\% }}.$$

Here, *W*_*1*_ and *W*_*2*_ refer to scratch width before and after ion insertion into the electrode, respectively.

Linear strain (*S*) measured by the SECM method was calculated using the following equation:

3 $${{S}} = \frac{{L_2 - L_1}}{{L_1}}.$$

Here, *L*_1_ and *L*_2_ are the thickness of the working electrode membrane before and after the test.

The displacement (*δ*) of the actuator was measured by a Keyence LK-G800 laser positioning system, where the strain (*ɛ*) and stress (*σ*) were estimated according to Eq. 4 and 5 in refs. ^17,59^:

4 $$\varepsilon = \frac{{2 \delta d}}{{\delta ^2 + L^2}},$$

5 $${\mathrm{\sigma }} = {\mathrm{\varepsilon }} {{Y}}.$$

Here, *d, L,* and *Y* are the thickness, free length, and Young’s modulus of the actuator, respectively.

Energy transduction efficiency of the actuator was calculated according to the following equation^64,65^:

6 $$\eta _{\mathrm{transduction}} = \frac{{P_{\mathrm{mechanical}}}}{{P_{\mathrm{input}}}} = \frac{{0.5Y \varepsilon ^2 f \cdot \mathrm{vol}}}{{I V}} \times 100{\mathrm{\% }}.$$

Here, *P*_input_ (W) and *P*_mechanical_ (W) are the input electric energy and output mechanical energy of the actuator, respectively. *I* (A) and *V* (V) refer to the current and voltage applied on the actuator. *Y* (Pa)*, ɛ* (%)*, f* (Hz), and vol (m^3^) refer to Young’s modulus, strain, voltage frequency, and volume of actuator, respectively.

### Data availability

The data that support the findings of this study are available from the corresponding author upon reasonable request.

## Electronic supplementary material

Supplementary Information
