# Supplementary material for: High-performance graphdiyne-based electrochemical actuators
Source: Nat Commun. 2018 Feb 21;9:752. doi: 10.1038/s41467-018-03095-1 (PMC5821823; doi:10.1038/s41467-018-03095-1)
Supplement: Supplementary file 1 — Supplementary Information [file 41467_2018_3095_MOESM1_ESM.pdf]

Supplementary Figures

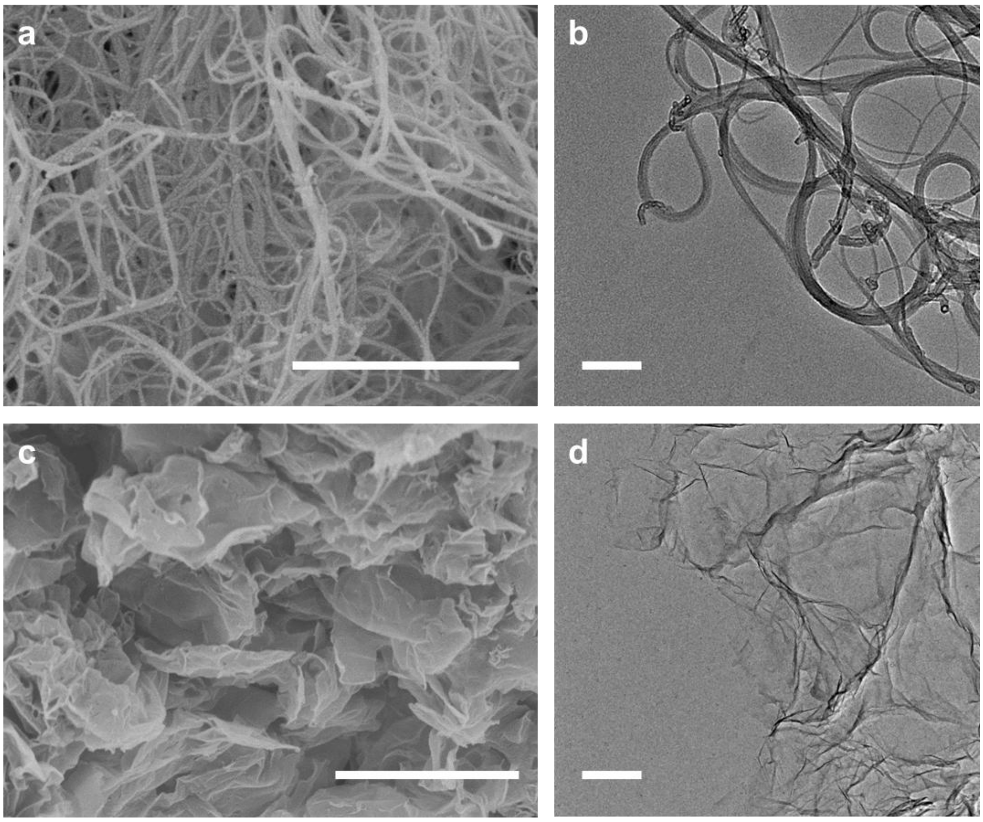

Supplementary Fig. 1 a SEM image of CNT, scale bar 1 um. **b** TEM image of CNT, scale bar 100 nm. **c** SEM image of graphene, scale bar 2 um. **d** TEM image of graphene, scale bar 200 nm.

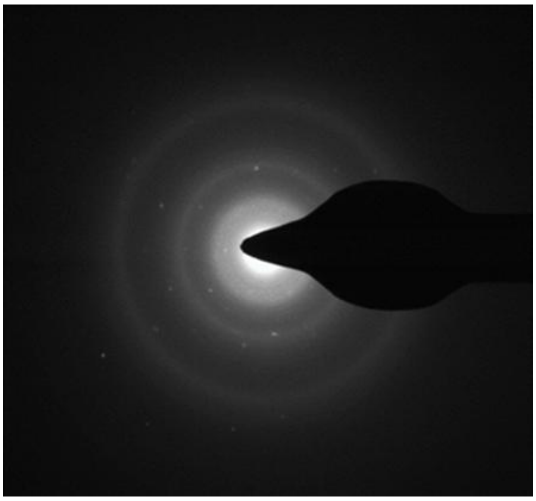

Supplementary Fig. 2 The selected-area electron diffraction pattern of graphdiyne.

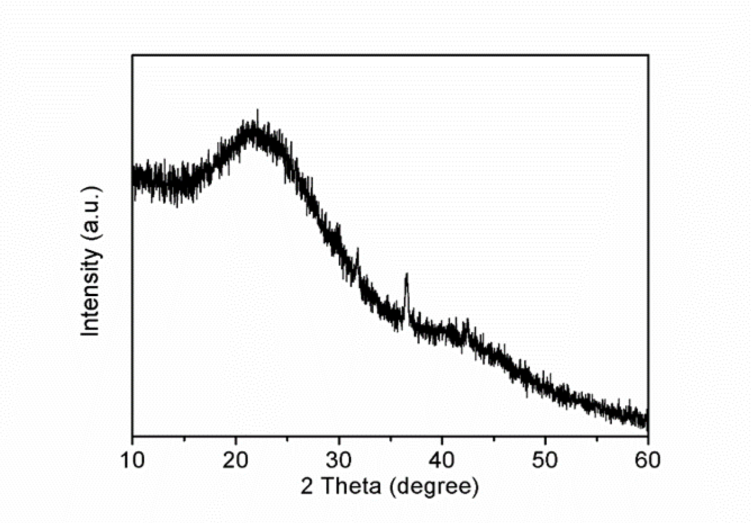

Supplementary Fig. 3 XRD pattern of graphdiyne powder.

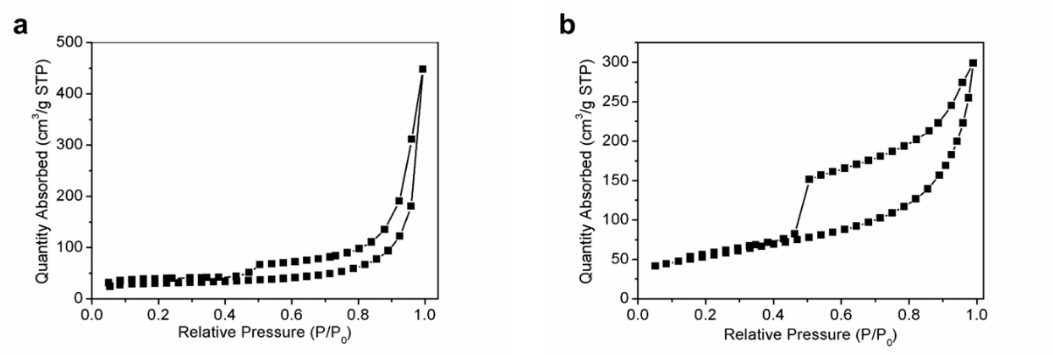

Supplementary Fig. 4 Nitrogen sorption isotherms of **a** CNT and **b** graphene, respectively.

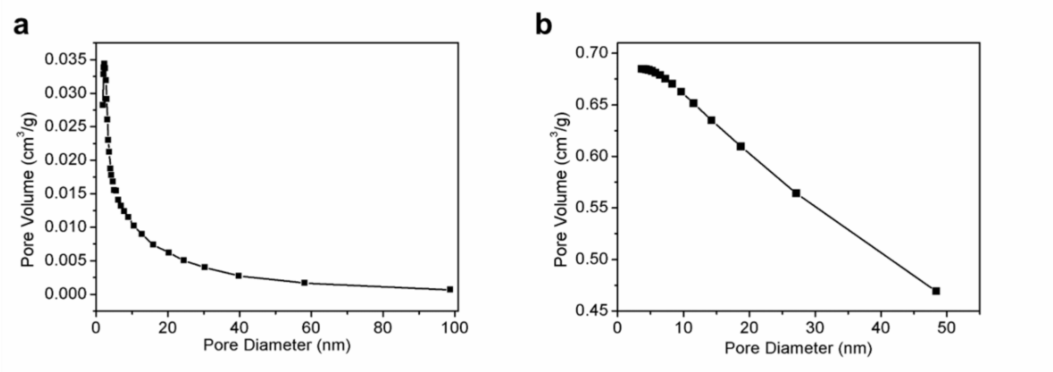

Supplementary Fig. 5 Pore size distribution of **a** CNT and **b** graphene, respectively.

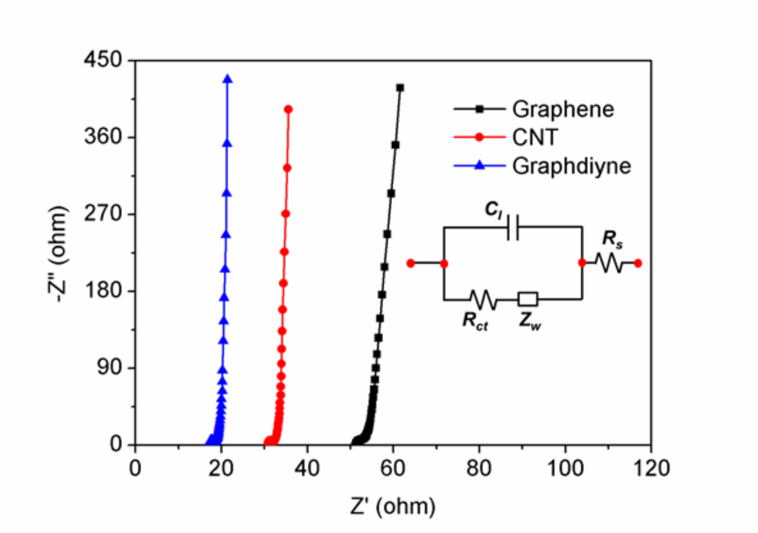

Supplementary Fig. 6 Nyquist plots for graphdiyne, graphene and CNT actuators. Inset is the equivalent circuit of devices.

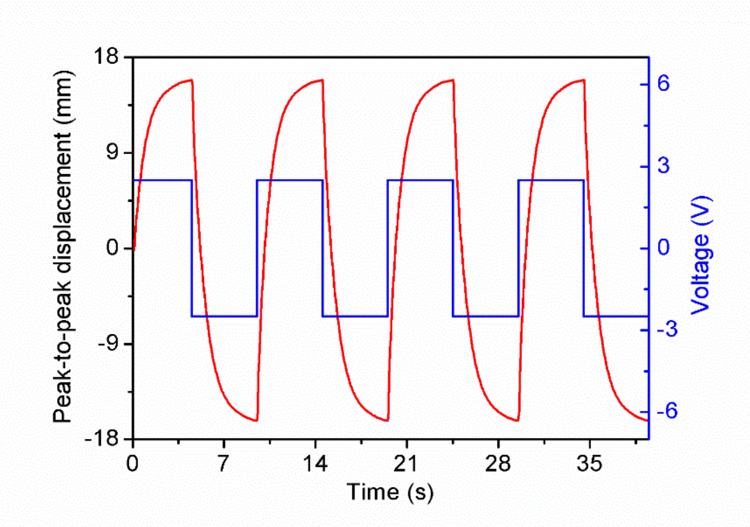

Supplementary Fig. 7 Actuation performance of graphdiyne actuator under 2.5 V at 0.1 Hz.

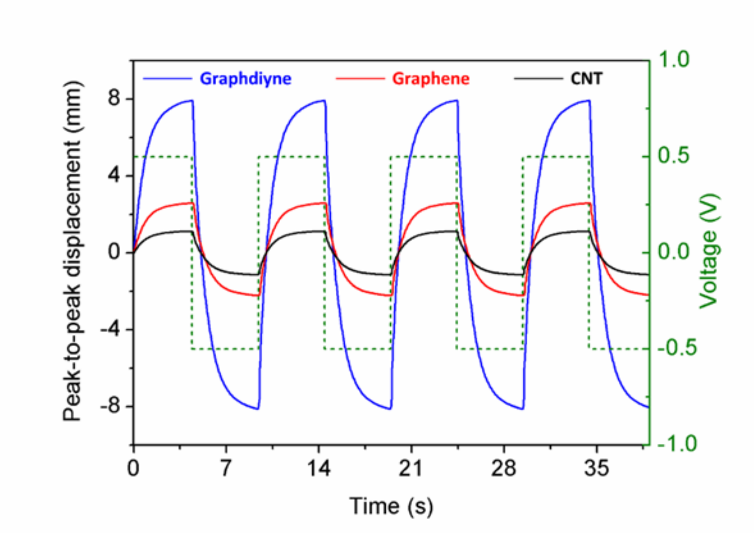

Supplementary Fig. 8 Comparison of the phase delay of all actuators under 0.5V at 0.1 Hz.

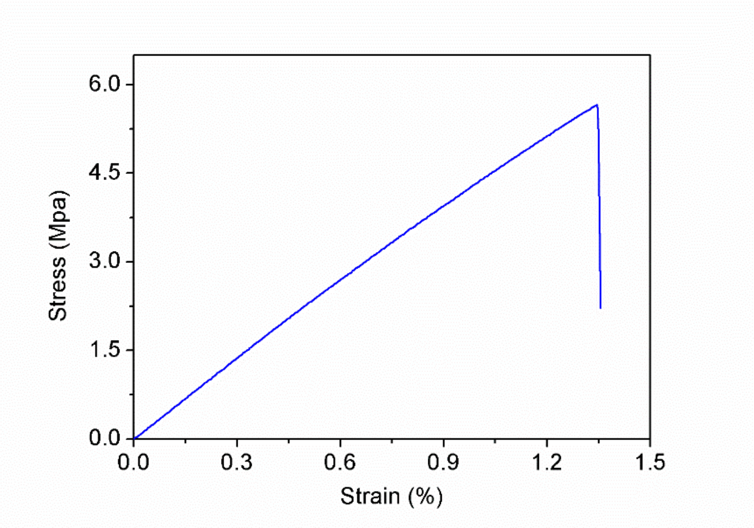

Supplementary Fig. 9 Stress-strain curve of graphdiyne actuator.

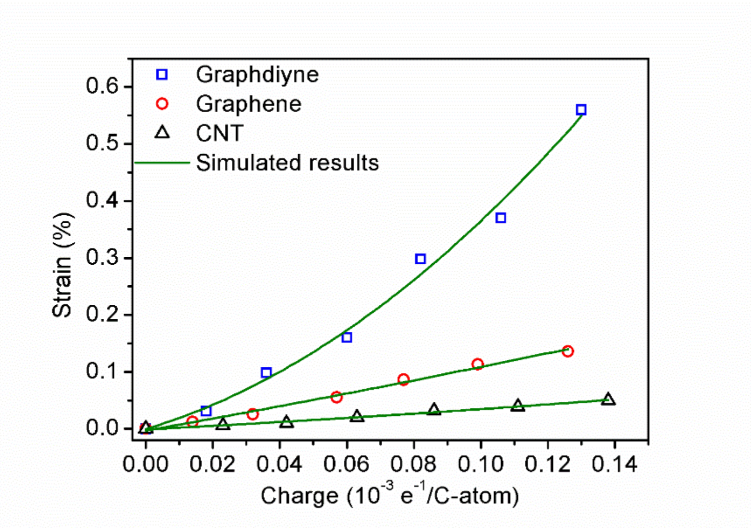

Supplementary Fig. 10 Contribution of charge injection to actuation strain of different actuators.

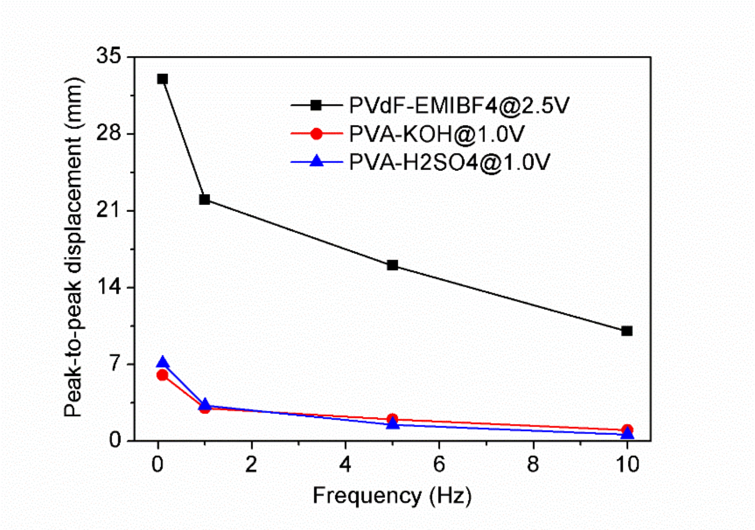

Supplementary Fig. 11 Comparison of actuation displacements of graphdiyne actuators based on PVA-H_2SO_4, PVA-KOH and PVdF-$EMIBF_4$ electrolyte, respectively.

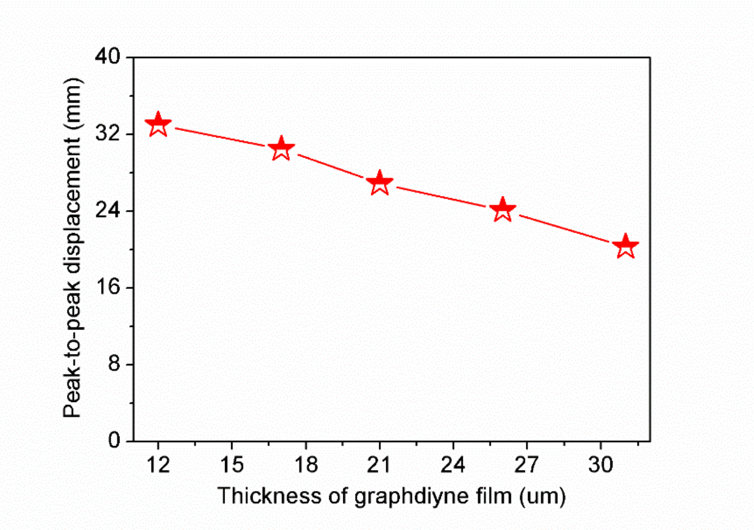

Supplementary Fig. 12 Effect of thickness of electrode films on the performance of graphdiyne actuator.

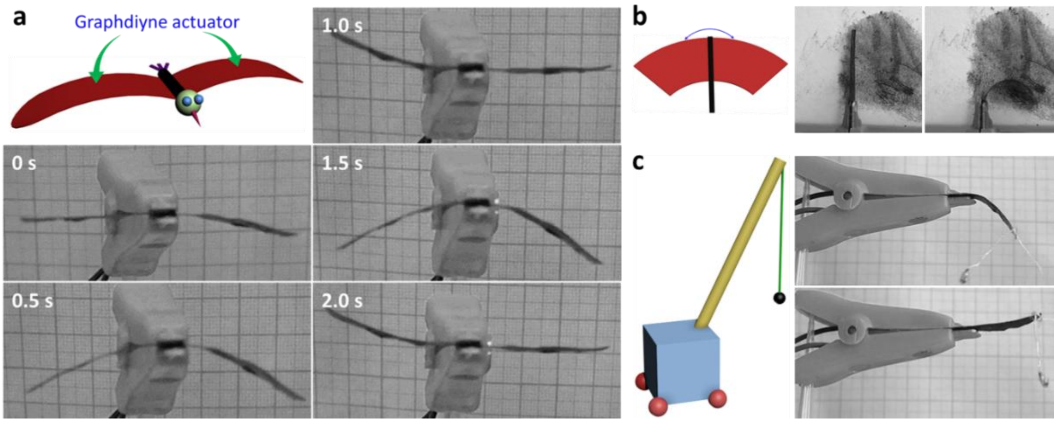

Supplementary Fig. 13 a Flying robot model. The robot starts flapping its wings under electric stimulus. **b** Wiper model. The wiper starts cleaning the floor with power on. **c** Crane model. The crane could lift the weight to a new position by controlling voltage.

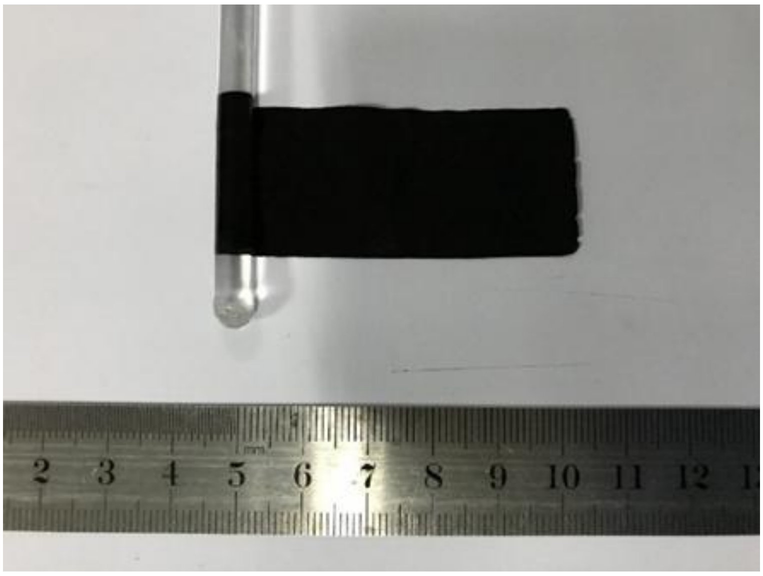

Supplementary Fig. 14 Photograph of freestanding graphdiyne film. Its left side is rolled around a glass rod to illustrate its good flexibility.

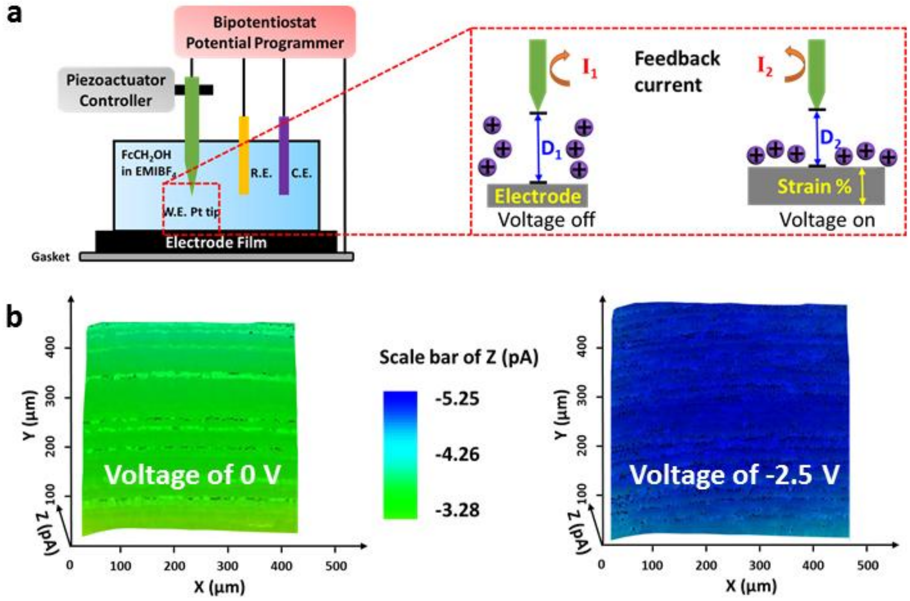

Supplementary Fig. 15 a Schematic presentation of the SECM system. **b** Schematic of the working process of SECM system. **c** Electrochemical images of graphdiyne electrode under the applied voltage of 0 V and -2.5 V.

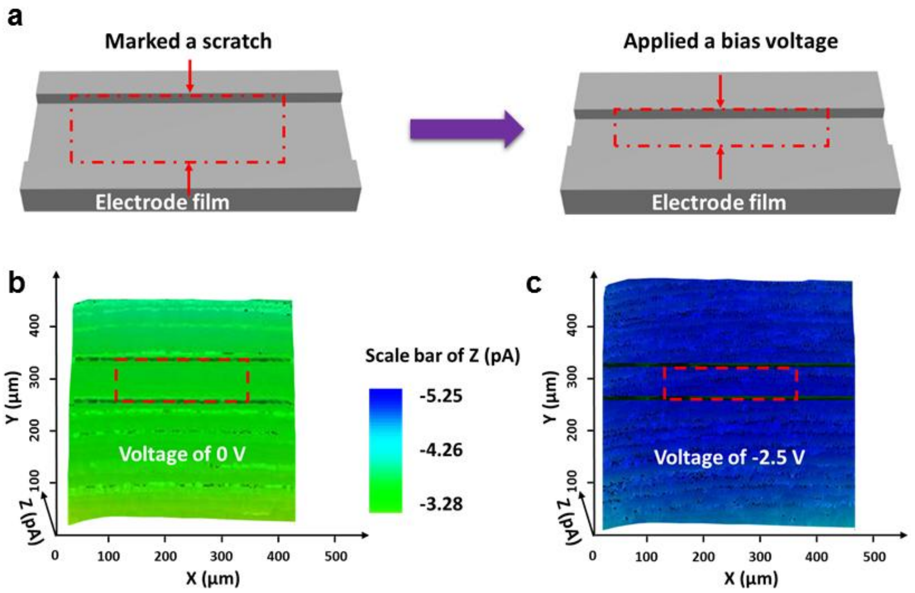

Supplementary Fig. 16 a Schematic illustration of the marked electrode by drawing a scratch and its related expansion process. **b** Electrochemical images of the marked electrode and **c** its related electromechanical expansion abilities under the applied voltage of -2.5 V, the dashed rectangular box with red lines were the marked nick.

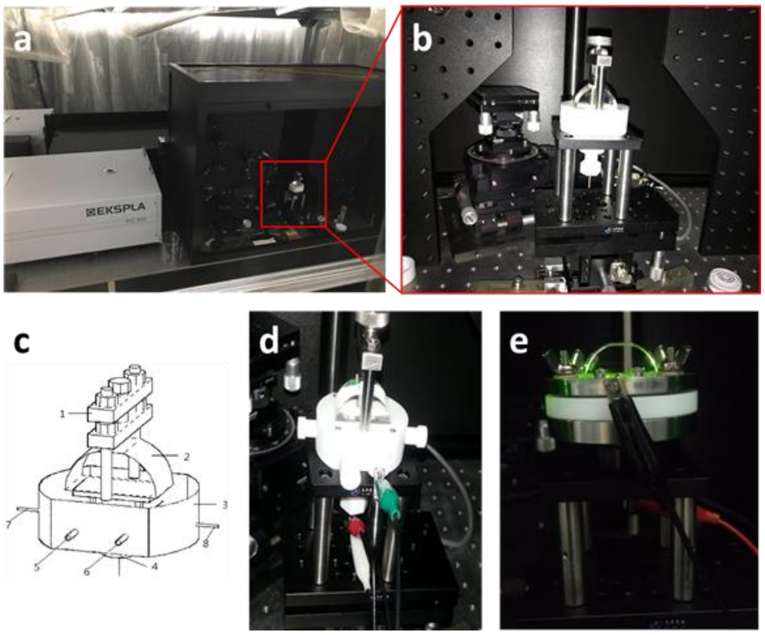

Supplementary Fig. 17 Photographs of in-situ SFG instrument. **a,** Overall view of SFG setup. **b,** Magnified chamber. **c,** Design sketch of SFG cell. (1. Adjustable lever 2. CaF_2 prism 3. Teflon cell 4. Working electrode 5. Counter electrode 6. Reference electrode 7, 8. Electrolyte conduit) **d,** Assembled SFG cell for test. **e,** SFG cell in test status.

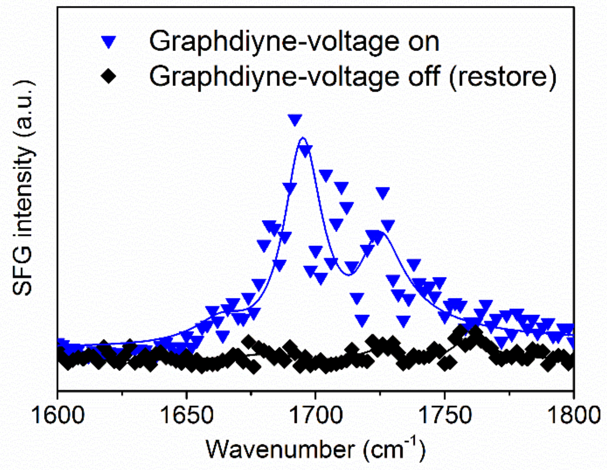

Supplementary Fig. 18 Recovery of SFG spectra at the alkene bond stretching region after cancelling electric stimulus.

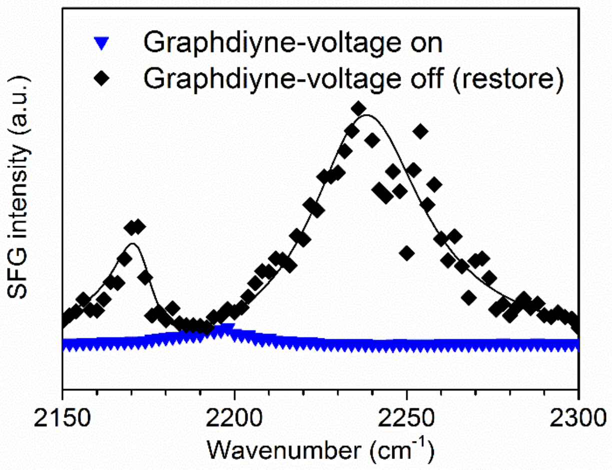

Supplementary Fig. 19 Recovery of SFG spectra at the alkyne bond stretching region after cancelling electric stimulus.

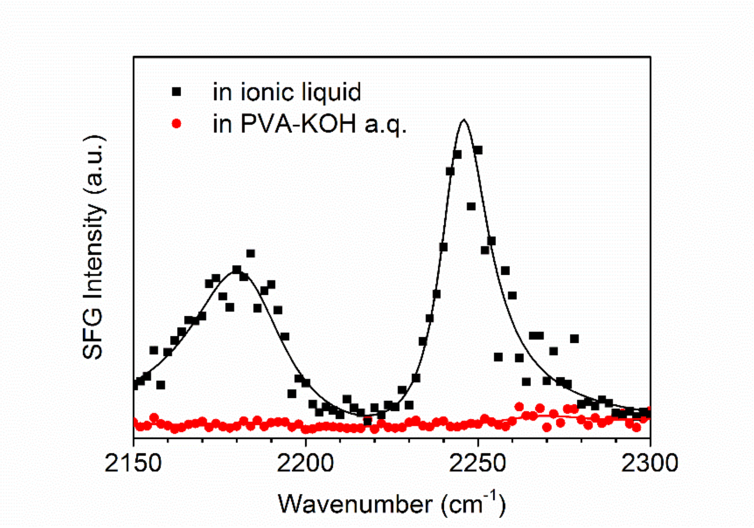

Supplementary Fig. 20 In-situ SFG measurements in aqueous gel electrolyte of PVA-KOH and ionic liquid electrolyte.

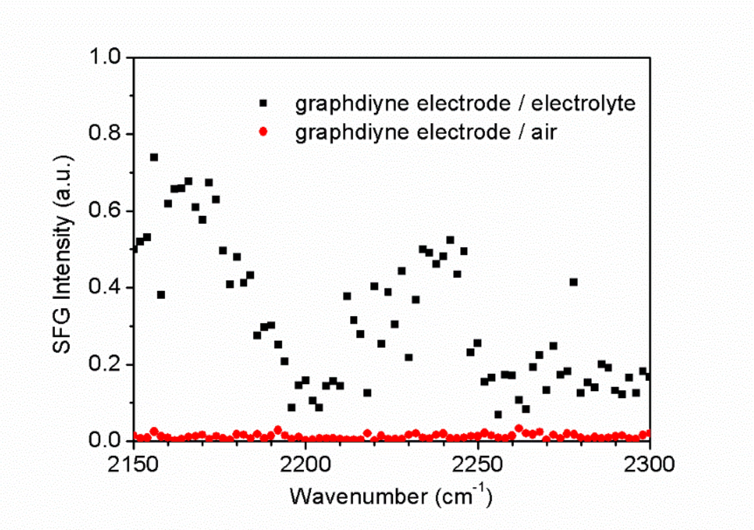

Supplementary Fig. 21 Comparison of SFG signal of graphdiyne electrode with and without electrolyte at the alkyne bond stretching region.

Supplementary Tables

Supplementary Table 1 EIS molding data.

Actuator type	R_s/ohm	R_{ct}/ohm	Z_w/ohm	C_I/F
CNT	32.1	24.58	3.39	0.46
Graphene	53.8	47.42	6.21	0.71
Graphdiyne	18.4	12.76	1.98	1.53

Supplementary Table 2 A comparative table on the actuation properties of graphdiyne with graphene, CNT and other materials.

	Strain (%)	Stress (MPa)	Blocking force (mN)	η_{trans} (%)
Graphdiyne (This work)	0.78	3.11	3.37	6.03
Graphene[1]	0.12	2.41	1.92	~0.1
CNT[2]	0.04	1.27	1.38	~0.1
Au/Pt[3]	0.02	2.48	—	0.1-0.2
CNT/Ppy[4]	~0.1	~0.65	—	—
MWNT/PEDOT[5]	0.64	3.76	1.43	1.04
MWNT/RuO_2[6]	0.95	1.01	—	—
Graphene/CNT[7]	~0.2	—	0.41	~0.1
VACNT/PANI[8]	0.22	—	—	—
Graphene/Ag[9]	0.05	—	—	~0.1
g-C_3N_4[10]	0.93	—	0.93	—
S-N co-doped graphene[11]	0.36	—	0.13	—

Supplementary Notes

Supplementary Note 1. Detailed mechanism of SFG method.

SFG is a second-order nonlinear process that is used to probe molecules at the interface between two media. The technique involves the overlap of two pulsed laser beams at the surface, generating a third beam with a frequency that is the sum of the two input frequencies. The SFG technique possesses inherent interface selectivity owing to the fact that even order susceptibilities become zero in centrosymmetric media under electric dipole approximation. The SFG intensity from an interface region is proportional to the incident laser intensities and the square of the absolute value of the effective sum frequency susceptibility $\chi_{\text{eff}}^{(2)}$[12], as shown in Supplementary Equation 1.

$$I(\omega) = \frac{8\pi^3\omega^2 \sec^2\beta}{c^3 n_1(\omega) n_1(\omega_1) n_1(\omega_2)} |\chi_{\text{eff}}^{(2)}|^2 I(\omega_1) I(\omega_2) \qquad (1)$$

where ω, ω_1, and ω_2 are the frequencies of the sum frequency signal, visible laser beam, and IR laser beam, respectively; $n_j(\omega_i)$ is the refractive index of bulk medium j at frequency ω_i; β is the reflection angle of the SFG light relative to the interface normal, which depends on the incident angles of the visible and IR laser beam according to the phase matching condition $n_j(\omega)\omega\sin\beta = n_j(\omega_1)\omega_1\sin\beta_1 + n_j(\omega_2)\omega_2\sin\beta_2$; $I(\omega)$, $I(\omega_1)$, and $I(\omega_2)$ are the intensities of the SFG signal, visible beam and IR beam, respectively.

SFG spectral fitting method and typical fitting results:

For SFG with contribution from multiple vibrational resonances, the SFG macroscopic susceptibility tensor $\chi_{ijk}^{(2)}$ can be written as Supplementary Equation 2[12].

$$\chi_{ijk}^{(2)} = \chi_{\text{NR},ijk}^{(2)} + \sum_q \frac{A_{q,ijk}}{\omega_2 - \omega_q + i\Gamma_q} \qquad (2)$$

$\chi_{\text{NR},ijk}^{(2)}$ represents the nonresonant contribution, and $A_{q,ijk}$, ω_q, and Γ_q are the oscillator strength, resonant frequency, and damping coefficient (line width in unit of circular frequency) of the qth vibrational mode, respectively.

SFG spectrum is recorded with certain polarization combinations. Polarizations are listed in order of SFG, visible beam, and IR beam, such that ssp polarization refers to S-polarized SFG signal generated from the incident S-polarized visible and P-

polarized IR beams. The SFG intensity (normalized by the incident intensities) in a particular polarization combination can be expressed in the following form[12-14]:

$$I(\omega_{\mathrm{IR}}) = C + B\left|\chi_{\mathrm{NR}}^{(2)} + \sum_q \frac{A_q}{\omega_{\mathrm{IR}} - \omega_q + i\Gamma_q}\right|^2 \qquad (3)$$

where *B* and *C* are two fitting constants.

In the present work, SFG spectra are recorded with ssp polarization combination. The experimental SFG spectra are fitted to Supplementary Equation 3, where *B* is normalized to unity for simplification, and *C* is limited to a small value to account for the drift of baseline.

To clarify the resonant contribution of each vibration mode, the fitting data were re-plotted after removing the non-resonant parts mathematically.

Supplementary Note 2. Specific analysis of peak assignment in graphdiyne.

Schematic of the SFG-VS electrochemical cell is shown in Fig. 5. Briefly, a thin layer of electrolyte ($EMIBF_4$) was sandwiched between a semicylinder CaF_2 prism and a graphdiyne electrode, and a stainless steel electrode was used as the counter. Two lasers with different frequencies was emitted onto the interface between electrolyte and electrode, and then SFG signal reflecting molecular structure information was collected by the detector. The polarization combination was set to ssp, which refers to S-polarized SFG signal generated from the incident S-polarized visible and P-polarized IR beams. Two resonant vibration modes can be recognized from the SFG spectra in the 2150-2300 cm^{-1} region. For the initial sample, two vibration bands peaked at 2180 cm^{-1} and 2246 cm^{-1} were observed in Supplementary Fig. 21. In previous works[15,16], it has been experimentally found that graphdiyne possesses a Raman peak at the 2180-2190 cm^{-1} range corresponding to the vibration of conjugated diyne links. On the other hand, theoretical simulation has pointed out graphdiyne has two Raman-active modes (2142 cm^{-1} and 2221 cm^{-1}) and one IR-active mode (2168 cm^{-1}) in the alkyne vibration region. Considering the centrosymmetric nature of the alkyne linkage, all these modes are expected to be SFG-inactive (SFG requires both Raman and IR activity). Indeed, pristine graphdiyne samples in the absence of the electrolyte yield no distinct resonant SFG response in the above region in Supplementary Fig. 21. We thus assign the observed SFG peaks for graphdiyne/$EMIBF_4$ to the vibration of the alkyne groups that are slightly complexed with the electrolyte. The complexation essentially breaks the centrosymmetricity of the alkyne moiety and shifts its vibration frequency as well. One previous report has demonstrated the appearance of new vibration modes in alkynes when being complexed with CF_3-X halogens. The new vibration modes are closely related to C≡C stretching and are both Raman and IR active[17]. The similar mechanism accounts for the complexation-induced SFG activity of alkyne in our case.

Supplementary References

1. Lu, L. et al. Highly stable air working bimorph actuator based on a graphene nanosheet/carbon nanotube hybrid electrode. *Adv. Mater.* **24**, 4317-4321 (2012).
2. Lu, L. & Chen, W. Biocompatible composite actuator: a supramolecular structure consisting of the biopolymer chitosan, carbon nanotubes, and an ionic liquid. *Adv. Mater.* **22**, 3745-3748 (2010).
3. Nemat-Nasser, S. Micromechanics of actuation of ionic polymer-metal composites. *J. Appl. Phys.* **92**, 2899-2915 (2002).
4. Mukai, K., Yamato, K., Asaka, K., Hata, K. & Oike, H. Actuator of double layer film composed of carbon nanotubes and polypyrroles. *Sens. Actuators, B* **161**, 1010-1017 (2012).
5. Wang, D. et al. High energy conversion efficiency conducting polymer actuators based on PEDOT:PSS/MWCNTs composite electrode. *RSC Adv.* **7**, 31264-31271 (2017).
6. Terasawa, N., Mukai, K., Yamato, K. & Asaka, K. Superior performance of non-activated multi-walled carbon nanotube polymer actuator containing ruthenium oxide over a single-walled carbon nanotube. *Carbon* **50**, 1888-1896 (2012).
7. Lu, L. et al. Highly stable air working bimorph actuator based on a graphene nanosheet/carbon nanotube hybrid electrode. *Adv. Mater.* **24**, 4317-4321 (2012).
8. Wu, G. et al. Ordered and Active Nanochannel Electrode Design for High-Performance Electrochemical Actuator. *Small* **12**, 4986-4992 (2016).
9. Lu, L., Liu, J., Hu, Y., Zhang, Y. & Chen, W. Graphene-stabilized silver nanoparticle electrochemical electrode for actuator design. *Adv. Mater.* **25**, 1270-1274 (2013).
10. Wu, G. et al. Graphitic carbon nitride nanosheet electrode-based high-performance ionic actuator. *Nat. Commun.* **6**, 7258-7265 (2015).
11. Kotal, M., Kim, J., Kim, K. J. & Oh, I. K. Sulfur and Nitrogen Co-Doped Graphene Electrodes for High-Performance Ionic Artificial Muscles. *Adv. Mater.* **28**, 1610-1615 (2016).
12. Zhuang, X., Miranda, P. B., Kim, D. & Shen, Y. R. Mapping molecular orientation and conformation at interfaces by surface nonlinear optics. *Phys Rev B* **59**, 12632-12640 (1999).
13. Lu, R., Gan, W., Wu, B. H., Chen, H. & Wang, H. F. Vibrational polarization spectroscopy of CH stretching modes of the methylene goup at the vapor/liquid interfaces with sum frequency

generation. *J Phys Chem B* **108**, 7297-7306 (2004).

14. Miranda, P. B. & Shen, Y. R. Liquid interfaces: A study by sum-frequency vibrational spectroscopy. *J Phys Chem B* **103**, 3292-3307 (1999).

15. Zhou, J. *et al.* Synthesis of Graphdiyne Nanowalls Using Acetylenic Coupling Reaction. *J. Am. Chem. Soc.* **137**, 7596-7599 (2015).

16. Li, G. *et al.* Architecture of graphdiyne nanoscale films. *Chem. Commun.* **46**, 3256-3258 (2010).

17. Nagels, N. & Herrebout, W. A. A cryospectroscopic infrared and Raman study of the CXcdots, three dots, centeredpi halogen bonding motif: complexes of the CF3Cl, CF3Br, and CF3I with ethyne, propyne and 2-butyne. *Spectrochimica acta. Part A, Molecular and biomolecular spectroscopy* **136**, 16-26 (2015).
